# Supplementary material for: Dynamic nucleosome organization after fertilization reveals regulatory factors for mouse zygotic genome activation
Source: Cell Res. 2022 Apr 15;32(9):801–13. doi: 10.1038/s41422-022-00652-8 (PMC9437020; doi:10.1038/s41422-022-00652-8)
Supplement: Supplementary file 1 — Supplementary information, Figure S1 [file 41422_2022_652_MOESM1_ESM.pdf]

Figure S1

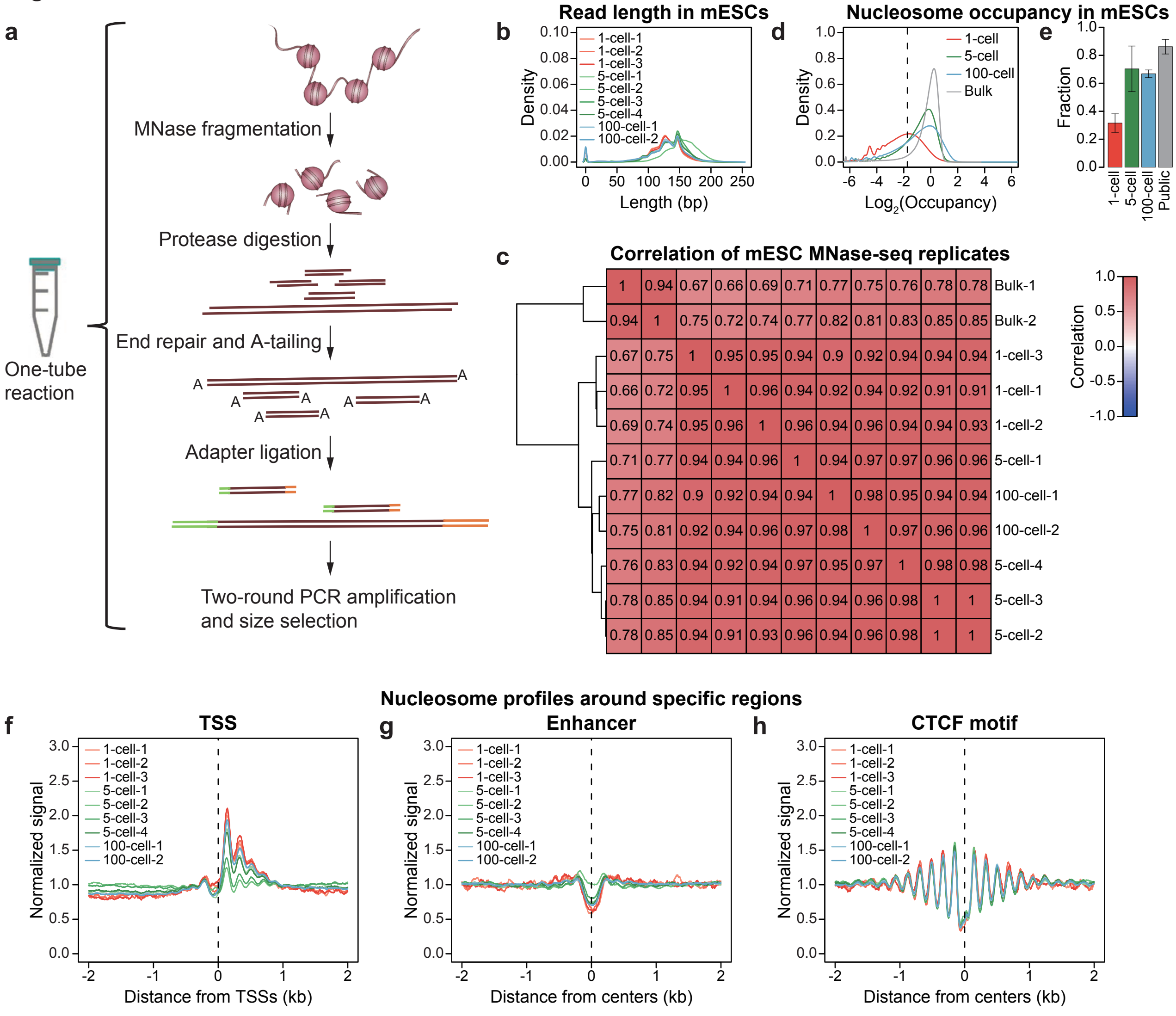

Fig. S1 Development of ULI-MNase-seq with mESC samples. a Schematic showing the procedures of ULI-MNase-seq. **b** Density plot showing the length distribution of mapped reads in mESC MNase-seq libraries started from different amounts of input. **c** Heatmap showing Pearson's correlation coefficients between MNase-seq replicates of mESC samples from different amounts of input, which were calculated based on the genome-wide nucleosome signal. **d** Density plot showing the distribution of the relative nucleosome occupancy of 1-kb bins in mESC samples from different amounts of input. The dashed line represents the cut-off for nucleosome-occupied regions (occupancy O > 0.3). **e** Bar plots showing the fraction of nucleosome-occupied 1-kb bins in mESC samples from different amounts of input. Error bars represent ±1.96*SD. **f, g and h** Nucleosome profiles around TSSs of Refseq genes (**f**), enhancers (**g**; defined using ESC ATAC-seq peaks) or CTCF motifs (**h**) in replicates of mESC samples from different amounts of input.
