# Supplementary material for: Dynamic nucleosome organization after fertilization reveals regulatory factors for mouse zygotic genome activation
Source: Cell Res. 2022 Apr 15;32(9):801–13. doi: 10.1038/s41422-022-00652-8 (PMC9437020; doi:10.1038/s41422-022-00652-8)
Supplement: Supplementary file 2 — Supplementary information, Figure S2 [file 41422_2022_652_MOESM2_ESM.pdf]

Figure S2

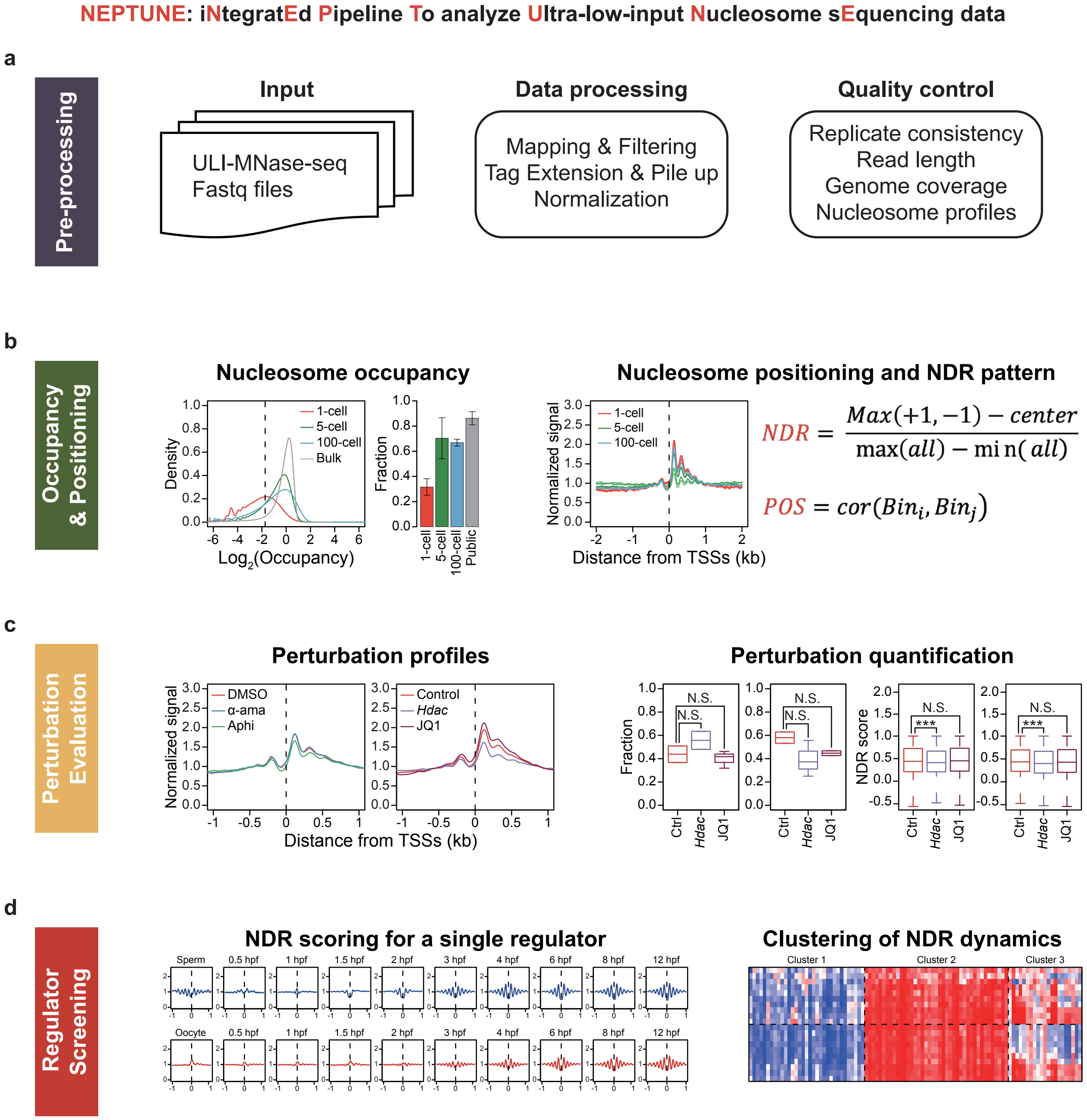

Fig. S2 NEPTUNE for integrated analysis of ULI-MNase-seq data. a Schematic diagram showing the preprocessing steps for the NEPTUNE pipeline. After mapping raw nucleosome datasets to the genome and filtering low-quality reads, NEPTUNE performs tag extension and piling up, and then normalizes the nucleosome signal according to sequence depth. NEPTUNE applies signal correlation analysis on replicates, read length analysis, genome coverage analysis and nucleosome profiling around specific regions as quality controls of ULI-MNase-seq data. **b** NEPTUNE estimates the nucleosome occupancy by taking the genome coverage of nucleosome reads and background noise into account. NEPTUNE also calculates nucleosome depletion scores (NDR scores) and phasing scores (POS scores) for summarizing the nucleosome positioning pattern. **c** NEPTUNE generates nucleosome profiles around TSSs of all genes or specific gene sets and calculates corresponding NDR scores as well as the genome-wide nucleosome coverage to evaluate the influence of different perturbations on nucleosome positioning. **d** NEPTUNE analyzes the dynamic change of NDR pattern on motif regions for individual regulators, and also classifies multiple regulators based on the NDR dynamics.
