# Supplementary material for: Dynamic nucleosome organization after fertilization reveals regulatory factors for mouse zygotic genome activation
Source: Cell Res. 2022 Apr 15;32(9):801–13. doi: 10.1038/s41422-022-00652-8 (PMC9437020; doi:10.1038/s41422-022-00652-8)
Supplement: Supplementary file 3 — Supplementary information, Figure S3 [file 41422_2022_652_MOESM3_ESM.pdf]

Figure S3

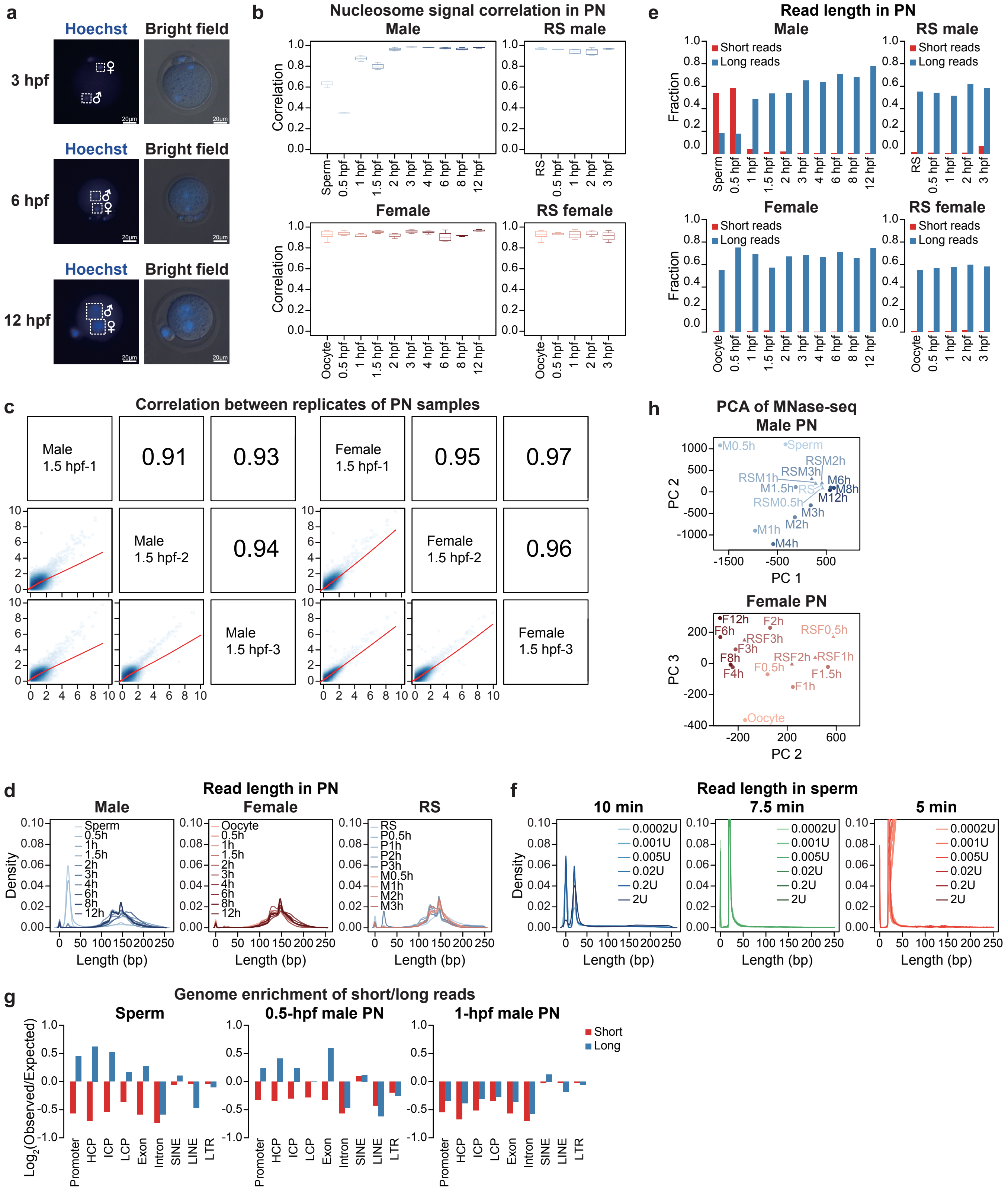

Fig. S3 Quality controls of ULI-MNase-seq in mouse pronuclei. a Illustration of the parental pronuclei at 3 hpf, 6 hpf and 12 hpf with Hoechst staining. **b** Boxplots showing the Pearson's correlation coefficients between MNase-seq replicates of each PN sample, which were calculated based on the nucleosome signal on promoter regions. **c** Scatter plots showing genome-wide nucleosome correlations between replicates of 1.5-hpf PN samples. **d** Density plots showing the length distribution of mapped reads in MNase-seq libraries at each PN stage. **e** Bar plots showing the fraction of short (5-50 bp) and long (120-180 bp) mapped reads in MNase-seq libraries at each PN stage. **f** Density plots showing the length distribution of mapped MNase-seq reads in sperm MNase-seq libraries under different conditions of MNase digestion. Color keys represent the amount of MNase used in each reaction, and subtitles indicate the duration of MNase digestion. **g** Bar plots showing the enrichment of short (5-50 bp) and long (120-180 bp) reads defined in sperm, 0.5-hpf or 1-hpf male PN on different genomic elements. **h** PCA analyses of ULI-MNase-seq samples at each PN stage. M, male PN. F, female PN. RSM, male PN of RS-fertilized embryos. RSF, female PN of RS-fertilized embryos. h, hpf.
