# Supplementary material for: Dynamic nucleosome organization after fertilization reveals regulatory factors for mouse zygotic genome activation
Source: Cell Res. 2022 Apr 15;32(9):801–13. doi: 10.1038/s41422-022-00652-8 (PMC9437020; doi:10.1038/s41422-022-00652-8)
Supplement: Supplementary file 4 — Supplementary information, Figure S4 [file 41422_2022_652_MOESM4_ESM.pdf]

Figure S4

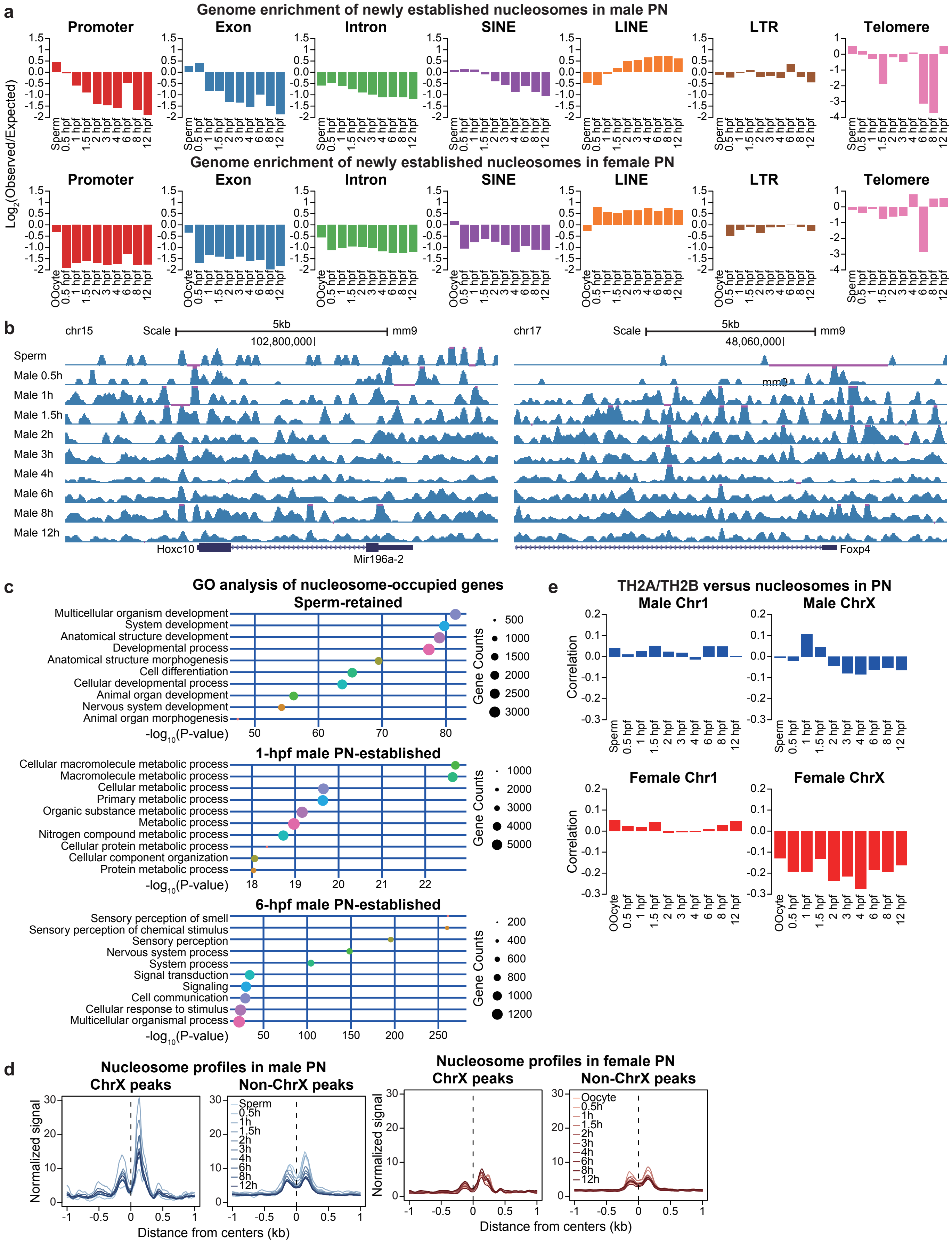

Fig. S4 Features of nucleosome occupancy in mouse pronuclei. a Bar plots showing the enrichment of newly established nucleosome regions defined at each PN stage on different genomic elements. **b** UCSC genome browser view of a sperm-retained nucleosome locus (left) and a locus with nucleosomes established in 1-hpf male PN (right). **c** GO analysis of genes with nucleosomes retained in sperm or newly established in 1-hpf/6-hpf male PN. **d** Nucleosome profiles around TH2A/TH2B peaks in X chromosomes or other chromosomes at each PN stage. **e** Bar plots showing the Pearson's correlation coefficients between nucleosome signals and TH2A/TH2B signals at each PN stage. h, hpf. Chr, chromosome.
