# Supplementary material for: Dynamic nucleosome organization after fertilization reveals regulatory factors for mouse zygotic genome activation
Source: Cell Res. 2022 Apr 15;32(9):801–13. doi: 10.1038/s41422-022-00652-8 (PMC9437020; doi:10.1038/s41422-022-00652-8)
Supplement: Supplementary file 5 — Supplementary information, Figure S5 [file 41422_2022_652_MOESM5_ESM.pdf]

Figure S5

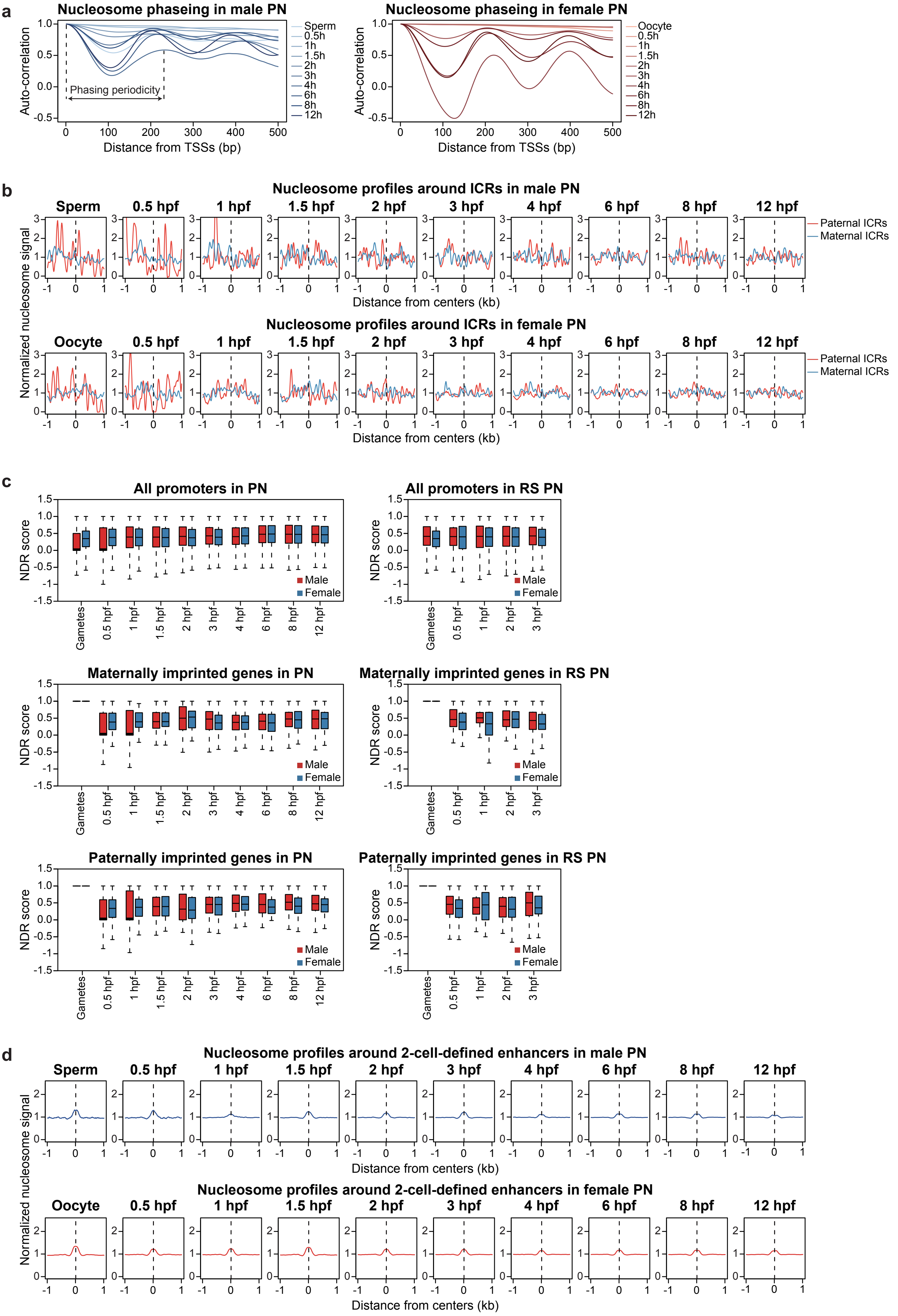
a
Nucleosome phaseing in male PN
Nucleosome phaseing in female PN
Auto-correlation
Phasing periodicity
Distance from TSSs (bp)
Sperm
0.5h
1h
1.5h
2h
3h
4h
6h
8h
12h
Oocyte
b
Nucleosome profiles around ICRs in male PN
Nucleosome profiles around ICRs in female PN
Sperm
Oocyte
0.5 hpf
1 hpf
1.5 hpf
2 hpf
3 hpf
4 hpf
6 hpf
8 hpf
12 hpf
Paternal ICRs
Maternal ICRs
Normalized nucleosome signal
Distance from centers (kb)
c
All promoters in PN
All promoters in RS PN
Maternally imprinted genes in PN
Maternally imprinted genes in RS PN
Paternally imprinted genes in PN
Paternally imprinted genes in RS PN
NDR score
Male
Female
Gametes
d
Nucleosome profiles around 2-cell-defined enhancers in male PN
Nucleosome profiles around 2-cell-defined enhancers in female PN

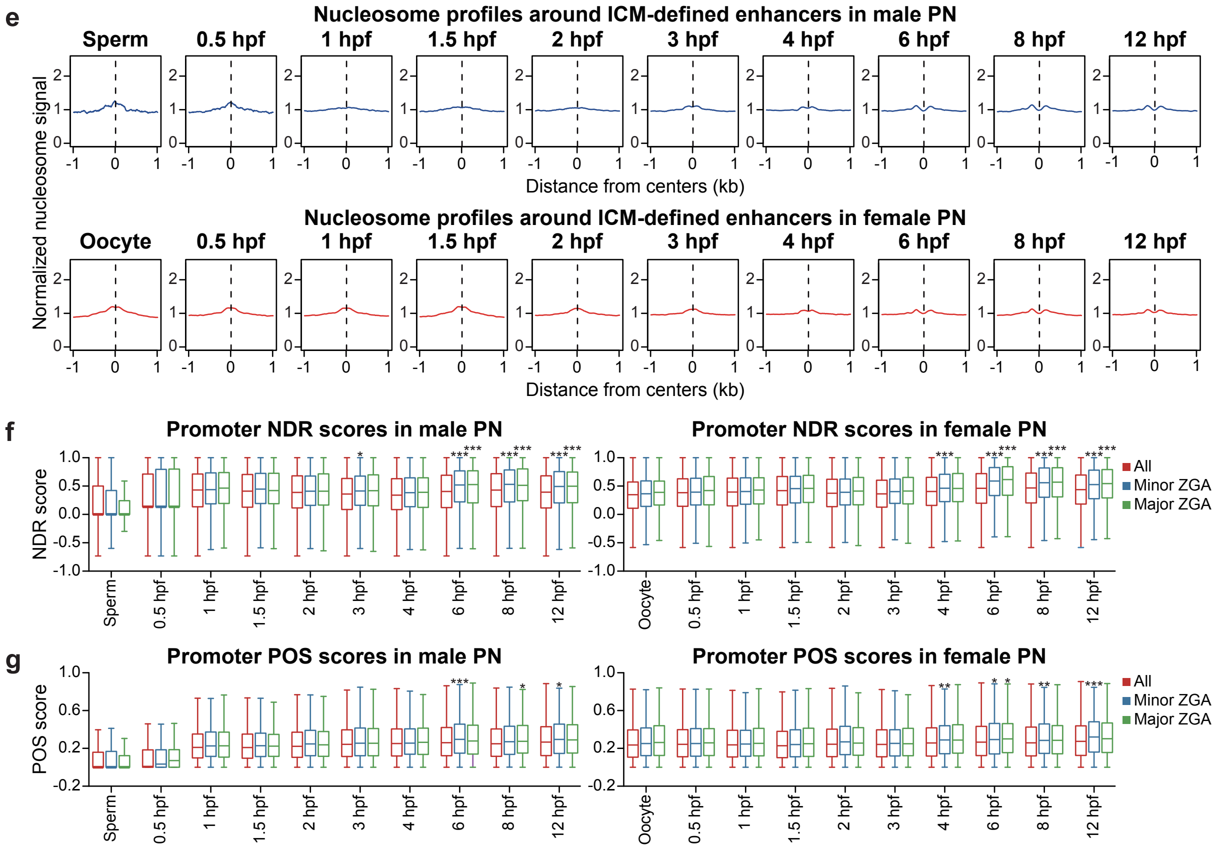

Fig. S5 Features of nucleosome positioning in mouse pronuclei. a Graph showing the auto-correlation of nucleosome signals downstream of TSSs at each PN stage. Y-axis represents the Pearson's correlation coefficient between the nucleosome signal of a designate site and the +10 bp site. Phasing periodicity (illustrated for 4-hpf male PN) represents the distance of the first peak summit from the TSS, and peaks within TSS +100 bp were ignored. h, hpf. **b** Nucleosome profiles around paternal and maternal imprinting control regions (ICRs) at each PN stage. **c** Boxplots showing NDR scores on promoters of all Refseq genes, maternally imprinted genes or paternally imprinted genes at each PN stage. **d and e** Nucleosome profiles around late 2-cell-defined (**d**) or ICM-defined (**e**) enhancers (defined using ATAC-seq peaks) at each PN stage. **f and g** Boxplots showing NDR scores (**f**) or POS scores (**g**) on promoters of all Refseq genes and ZGA genes at each PN stage. Significant difference is calculated between the designated gene set with all genes (*** $p < 0.001$; ** $p < 0.01$; * $p < 0.05$), and it is not labeled if $p > 0.05$.
