# Supplementary material for: Dynamic nucleosome organization after fertilization reveals regulatory factors for mouse zygotic genome activation
Source: Cell Res. 2022 Apr 15;32(9):801–13. doi: 10.1038/s41422-022-00652-8 (PMC9437020; doi:10.1038/s41422-022-00652-8)
Supplement: Supplementary file 6 — Supplementary information, Figure S6 [file 41422_2022_652_MOESM6_ESM.pdf]

Figure S6

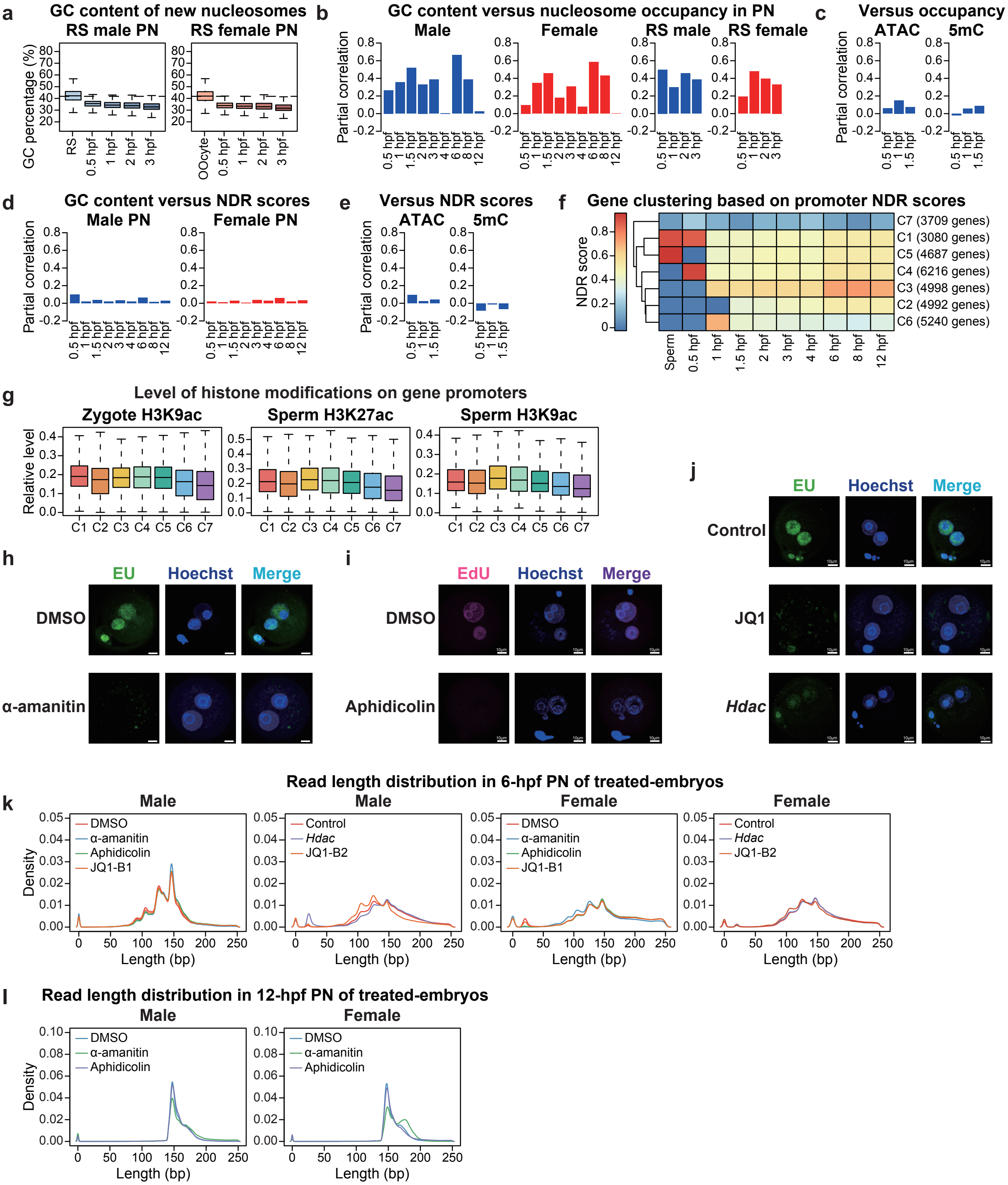

Fig. S6 Determinants of nucleosome occupancy and positioning in mouse pronuclei. a Boxplots showing the GC content of newly established nucleosome regions at each PN stage after ROSI-mediated fertilization. Dashed lines represent the average GC content in genome. **b and c** Bar plots showing the partial correlation between nucleosome occupancy and GC content (**b**), chromatin accessibility (**c** left; defined using sperm ATAC-seq peaks), or DNA methylation (**c** right; defined using sperm whole-genome bisulfite sequencing data) at each PN stage. **d and e** Bar plots showing the partial correlation between promoter NDR scores and GC content (**d**), chromatin accessibility (**e** left; defined same with c left) or DNA methylation (**e** right; defined as c right) at each PN stage. **f** Heatmap showing the k-means clustering (k=7) of coding genes based on promoter NDR scores at each male PN stage. **g** Boxplots showing the level of indicated histone modifications on promoters of genes in different promoter NDR clusters (defined in **f**). Y-axis represents the normalized ChIP-seq signals. **h, i and j** Confocal microscopy images of 12-hpf treated embryos after EU (**h** and **j**) or EdU (**i**) staining. **k, and l** Density plots showing the length distribution of mapped reads in MNase-seq libraries of 6-hpf (**k**) or 12-hpf (**l**) parental PN from groups under different treatment. JQ1-B1, JQ1 batch1. JQ1-B2, JQ1 batch2.
