# Supplementary material for: Dynamic nucleosome organization after fertilization reveals regulatory factors for mouse zygotic genome activation
Source: Cell Res. 2022 Apr 15;32(9):801–13. doi: 10.1038/s41422-022-00652-8 (PMC9437020; doi:10.1038/s41422-022-00652-8)
Supplement: Supplementary file 7 — Supplementary information, Figure S7 [file 41422_2022_652_MOESM7_ESM.pdf]

Figure S7

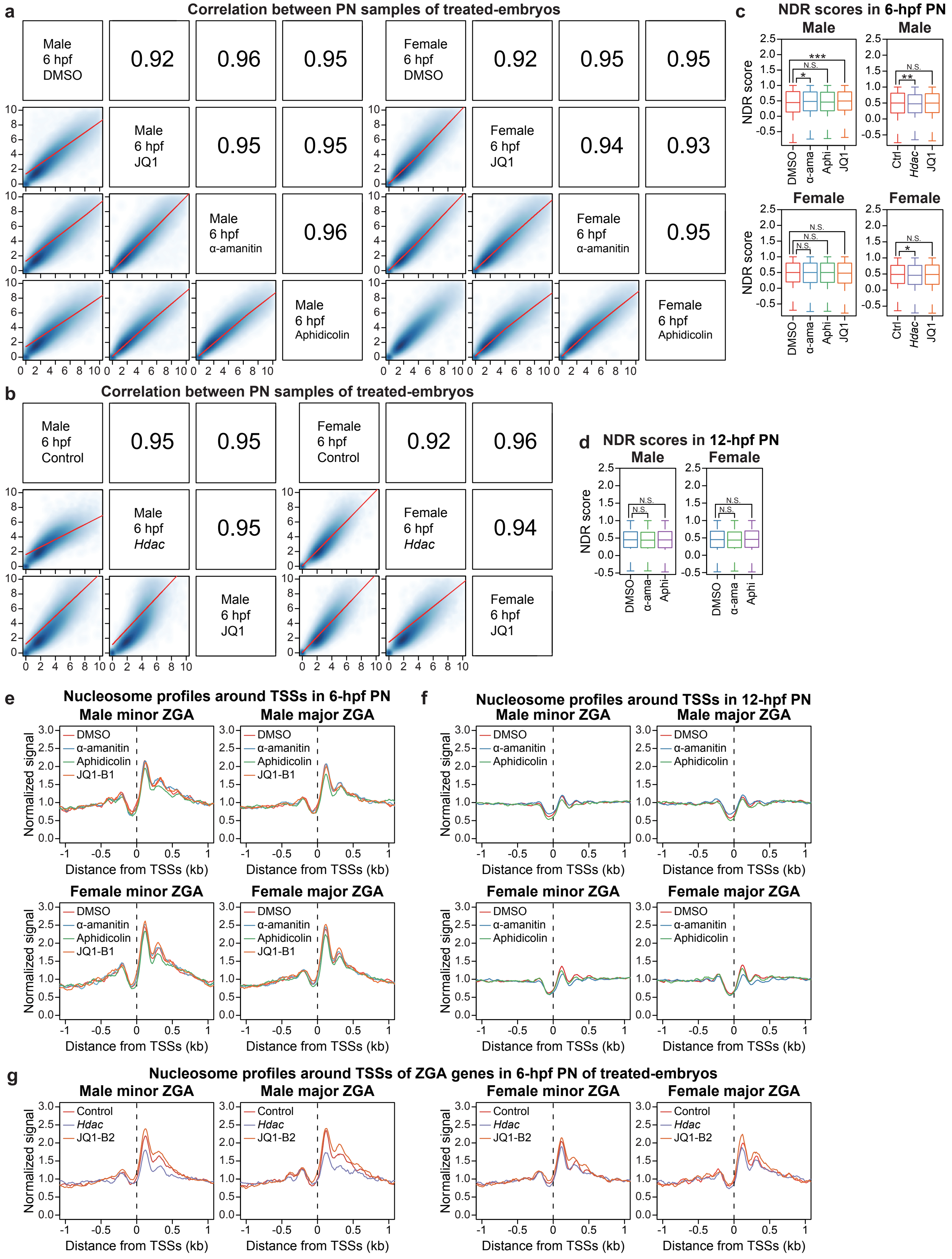

Fig. S7 Histone acetylation affects NDR establishment in mouse male pronuclei. a and b Scatter plots showing genome-wide nucleosome correlations between 6-hpf PN samples under different treatment. The first (**a**) and the second (**b**) batch of data were compared separately. c and d Boxplots showing the promoter NDR scores in 6-hpf (**c**) or 12-hpf (**d**) parental PN from groups under different treatment (*** $p < 0.001$; N.S. $p > 0.05$). **e, f and g** Nucleosome profiles around TSSs of ZGA genes in 6-hpf (**e and g**) or 12-hpf (**f**) parental PN from groups under different treatment. JQ1-B1, JQ1 batch1. JQ1-B2, JQ1 batch2.
