# Supplementary material for: Dynamic nucleosome organization after fertilization reveals regulatory factors for mouse zygotic genome activation
Source: Cell Res. 2022 Apr 15;32(9):801–13. doi: 10.1038/s41422-022-00652-8 (PMC9437020; doi:10.1038/s41422-022-00652-8)
Supplement: Supplementary file 8 — Supplementary information, Figure S8 [file 41422_2022_652_MOESM8_ESM.pdf]

Figure S8

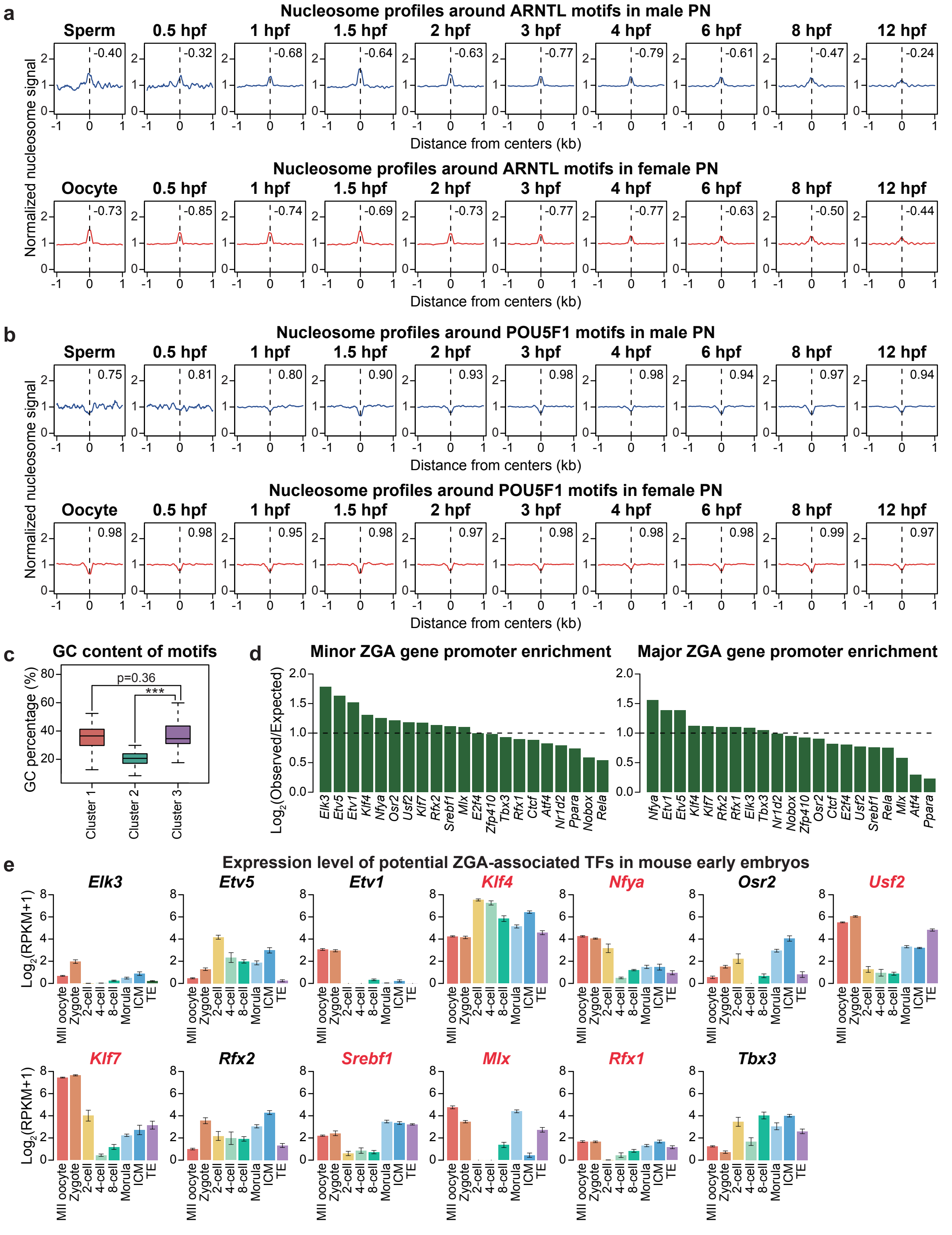

Fig. S8 Screening for potential ZGA-associated transcription factors. a and b Nucleosome profiles around ARNTL (**a**) or POU5F1 (**b**) motifs at each PN stage. NDR scores on motif regions calculated based on the averaged nucleosome profile of each stage are labeled. **c** Boxplot showing the GC content of motifs of TFs in different clusters (defined in **Fig. 4b**; *** $p < 0.001$). **d** Bar plots showing the enrichment of ZGA gene promoters on motifs of individual cluster-3 TFs (defined in **Fig. 4b**). **e** Bar plots showing the expression level of potential ZGA-associated TFs during mouse preimplantation development. Error bars represent ±1.96*SD.
