# Supplementary material for: Dynamic nucleosome organization after fertilization reveals regulatory factors for mouse zygotic genome activation
Source: Cell Res. 2022 Apr 15;32(9):801–13. doi: 10.1038/s41422-022-00652-8 (PMC9437020; doi:10.1038/s41422-022-00652-8)
Supplement: Supplementary file 9 — Supplementary information, Figure S9 [file 41422_2022_652_MOESM9_ESM.pdf]

Figure S9

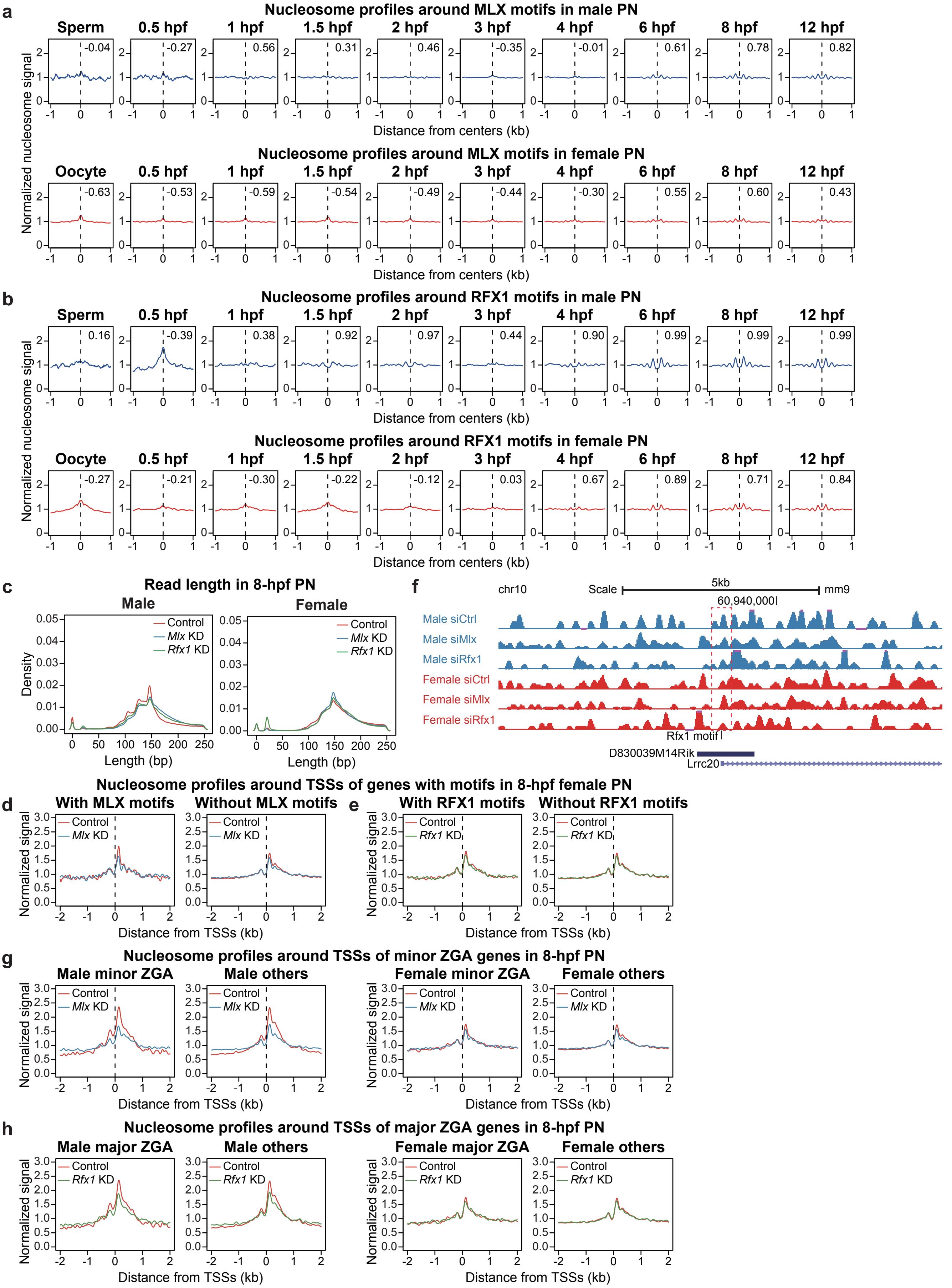

Fig. S9 Failure of promoter NDR establishment in male PN after Mlx or Rfx1 silencing. a and b Nucleosome profiles around MLX (**a**) or RFX1 (**b**) motifs at each PN stage. NDR scores of individual stages are labeled. **c** Density plots showing the length distribution of mapped reads in MNase-seq libraries of 8-hpf parental PN from KD groups. **d and e** Nucleosome profiles around TSSs of different classes of genes in 8-hpf maternal PN from KD groups. Genes were classified according to whether motifs of MLX (**d**) or RFX1 (**e**) are present in the promoters. **f** UCSC genome browser view of an RFX1 motif-containing site with a decrease in +1 nucleosomes after Rfx1 knockdown in both male and female PN. **g and h** Nucleosome profiles around TSSs of different classes of genes in 8-hpf parental PN from KD groups. Genes were classified according to whether they are minor (**g**) or major (**h**) ZGA genes.
