# Supplementary material for: Dynamic nucleosome organization after fertilization reveals regulatory factors for mouse zygotic genome activation
Source: Cell Res. 2022 Apr 15;32(9):801–13. doi: 10.1038/s41422-022-00652-8 (PMC9437020; doi:10.1038/s41422-022-00652-8)
Supplement: Supplementary file 10 — Supplementary information, Figure S10 [file 41422_2022_652_MOESM10_ESM.pdf]

Figure S10

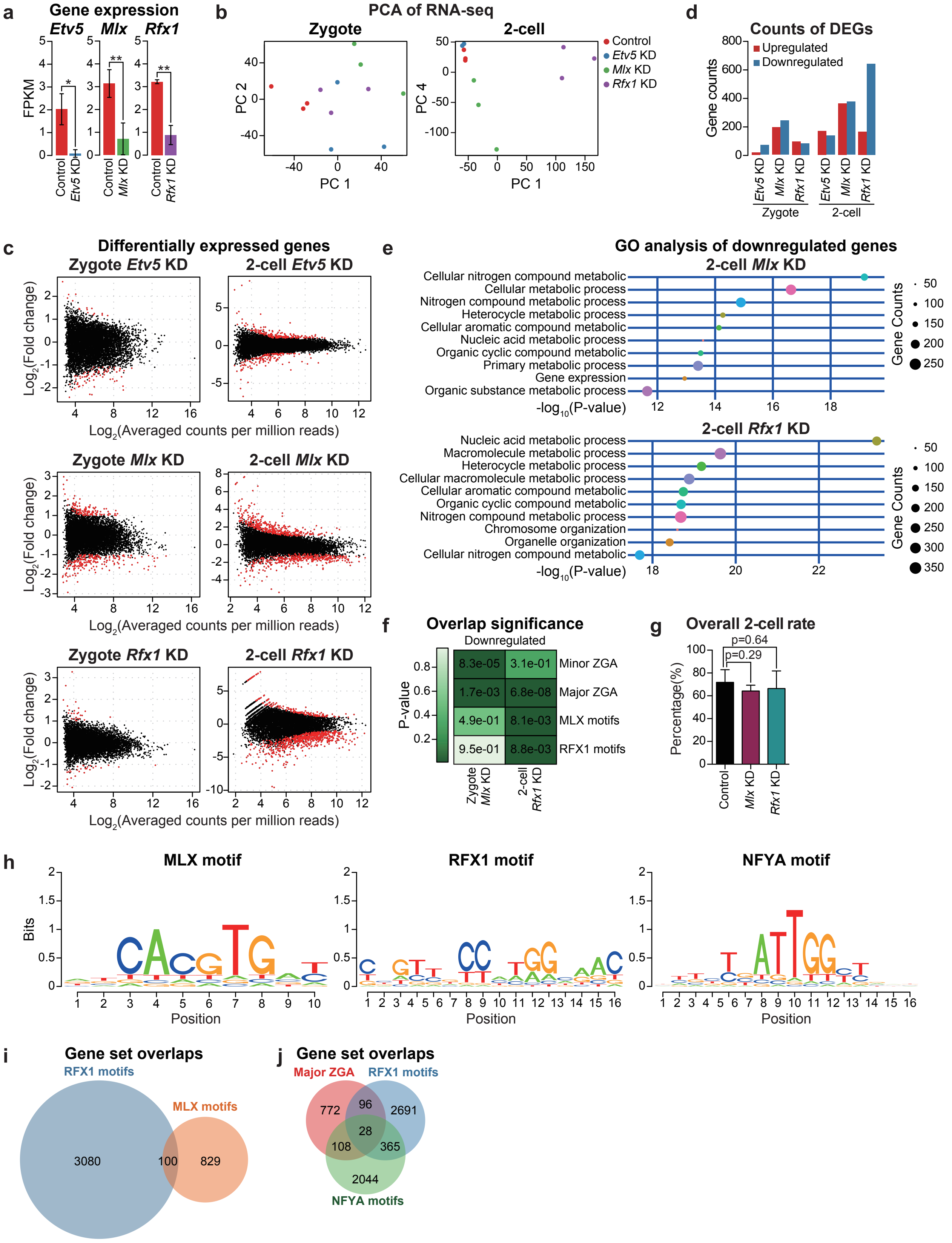

Fig. S10 Mlx or Rfx1 silencing blocks the ZGA process in mouse embryos. a Bar plots showing the expression level of siRNA-targeted genes in KD embryos (** $p < 0.01$; * $p < 0.05$). Error bars represent ±1.96*SD. **b** PCA analyses of RNA-seq replicates from KD groups. **c** Scatter plots showing the averaged expression level of genes (x-axis) and the fold change of genes upon KD (y-axis). Differentially expressed genes are labeled in red. **d** Bar plots showing counts of differentially expressed genes in KD embryos. **e** GO analysis of downregulated genes in Mlx or Rfx1 KD 2-cell embryos. **f** Heatmap showing the significance of overlaps (calculated as p-values of the hypergeometric test) between downregulated genes in KD embryos and indicated gene sets. **g** Bar plot showing the overall 2-cell rates of KD groups. n=3 biological replicates with approximately 30 embryos each. **h** Sequence logo representing the deduced consensus motif of MLX, RFX1 and NFYA. **i** Venn diagram showing the overlap between genes with RFX1 motifs and genes with MLX motifs. **j** Venn diagram showing the overlap between major ZGA genes, genes with NFYA motifs and genes with RFX1 motifs.
